# 9-[4-Hydr­oxy-3-(hydroxy­meth­yl)but­yl]guanine monohydrate

**DOI:** 10.1107/S1600536809043980

**Published:** 2009-10-28

**Authors:** Huang Tang, Feng-Jie Cheng, Nan Li, Yan-Cheng Liu, Zhen-Feng Chen

**Affiliations:** a Key Laboratory for the Chemistry and Molecular Engineering of Medicinal Resources (Ministry of Education), School of Chemistry & Chemical Engineering, Guangxi Normal University, Guilin 541004, People’s Republic of China

## Abstract

In the mol­ecular structure of the title compound, also named penciclovir monohydrate, C_10_H_15_N_5_O_3_·H_2_O, the 4-hydr­oxy-3-hydroxy­methyl­but-1-yl group is connected to guanine through an N atom of the imidazole ring. Water mol­ecules stabilize the mol­ecular packing by forming O—H⋯O hydrogen bonds. A three-dimensional network is generated *via* inter­molecular N—H⋯N, N—H⋯O, O—H⋯N and O—H⋯O hydrogen bonding.

## Related literature

For the synthesis and biological properies of penciclovir, see: Harnden & Jarvest (1985*a* ▶,*b* ▶); Hodge *et al.*(1989 ▶); Boyd *et al.* (1987 ▶). For the medicinal applications of penciclovir, see: Abdel-Hag *et al.* (2006 ▶); Andrei *et al.* (2004 ▶); Schmid-Wendtner & Korting (2004 ▶); Smith *et al.* (2001 ▶). 
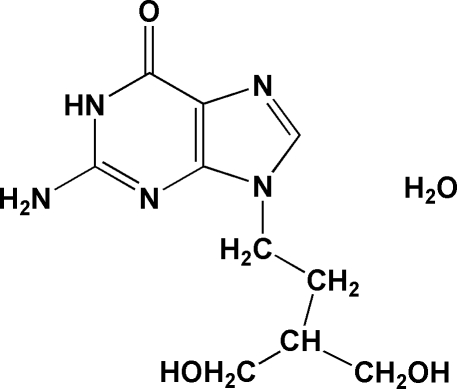


## Experimental

### 

#### Crystal data

C_10_H_15_N_5_O_3_·H_2_O
*M* *_r_* = 271.29
Orthorhombic, 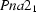

*a* = 8.2020 (16) Å
*b* = 13.889 (3) Å
*c* = 11.001 (2) Å
*V* = 1253.2 (4) Å^3^
*Z* = 4
Mo *K*α radiation
μ = 0.11 mm^−1^
*T* = 293 K
0.54 × 0.45 × 0.08 mm

#### Data collection

Bruker SMART CCD area-detector diffractometer
Absorption correction: multi-scan (*SADABS*; Sheldrick, 2001 ▶) *T*_min_ = 0.957, *T*_max_ = 0.994
6830 measured reflections
1193 independent reflections
1084 reflections with *I* > 2σ(*I*)
*R*_int_ = 0.075

#### Refinement

*R*[*F* ^2^ > 2σ(*F* ^2^)] = 0.050
*wR*(*F* ^2^) = 0.118
*S* = 1.06
1193 reflections
186 parameters
4 restraints
H atoms treated by a mixture of independent and constrained refinement
Δρ_max_ = 0.15 e Å^−3^
Δρ_min_ = −0.31 e Å^−3^

### 

Data collection: *SMART* (Bruker, 2001 ▶); cell refinement: *SAINT-Plus* (Bruker, 2001 ▶); data reduction: *SAINT-Plus*; program(s) used to solve structure: *SHELXS97* (Sheldrick, 2008 ▶); program(s) used to refine structure: *SHELXL97* (Sheldrick, 2008 ▶); molecular graphics: *SHELXTL* (Sheldrick, 2008 ▶); software used to prepare material for publication: *SHELXTL*.

## Supplementary Material

Crystal structure: contains datablocks I, global. DOI: 10.1107/S1600536809043980/ds2005sup1.cif

Structure factors: contains datablocks I. DOI: 10.1107/S1600536809043980/ds2005Isup2.hkl

Additional supplementary materials:  crystallographic information; 3D view; checkCIF report

## Figures and Tables

**Table 1:** Hydrogen-bond geometry (Å, °)

*D*—H⋯*A*	*D*—H	H⋯*A*	*D*⋯*A*	*D*—H⋯*A*
O2—H2⋯O4^i^	0.82	1.90	2.719 (3)	175
O3—H3*A*⋯N3^ii^	0.82	2.24	3.052 (3)	169
N4—H4⋯N2^iii^	0.96 (3)	1.86 (3)	2.816 (3)	176 (3)
O4—H4*A*⋯O2^iv^	0.86 (3)	1.93 (3)	2.787 (3)	178 (3)
O4—H4*B*⋯O1^v^	0.84 (3)	2.11 (5)	2.842 (3)	146 (3)
N5—H5*A*⋯O2^i^	0.86	2.15	2.898 (3)	146
N5—H5*B*⋯O1^iii^	0.86	2.11	2.931 (3)	159

Symmetry codes: (i) 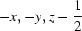; (ii) 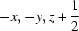; (iii) 
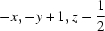
; (iv) 
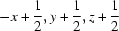
; (v) 
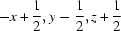
.

**Fig. 1.**
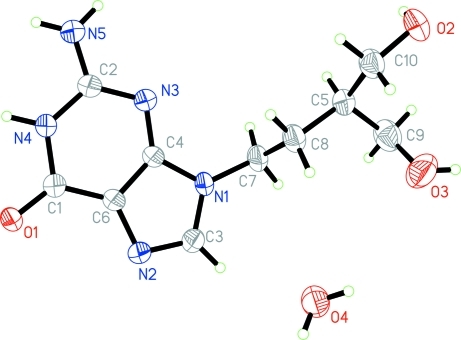
The molecular structure of (I),showing the atom-labelling scheme. Displacement ellipsoids are drawn at the 50% probability level.

**Fig. 2.**
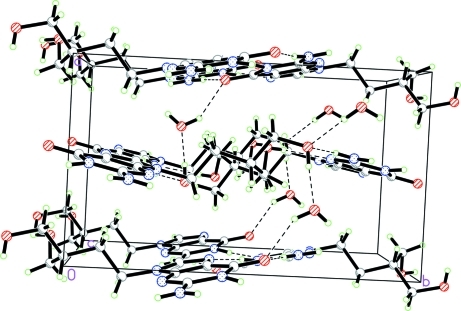
Three-dimensional structure of (I) along [001] direction. Hydrogen bonds are shown as dashed lines.

## supplementary crystallographic information

### Comment

9-[4-Hydroxy-3-(hydroxymethyl)butyl]guanine (I), known as penciclovir, is very effective for the treatment of herpes simplex virus, varicella zoster virus, Epstein–Barr virus, hepatitis virus and cytomegalovirus (Abdel-Hag *et al.*, 2006; Andrei *et al.*, 2004; Schmid-Wendtner and Korting, 2004; Smith *et al.*, 2001). The crystal lattice is built from molecules of (I) and waters of crystallization (Fig. 1). The guanine ring in (I) is coplanar wherein the C—N bond distances range from 1.312 (4) to 1.395 (5) Å. Three dimensional network is generated *via* N—H···N, N—H···O (2.816 (3)–2.931 (3) Å,), O—H···N(3.052 (3) Å), and O—H···O(2.719 (3)–2.842 (3) Å) hydrogen bonds from water and penciclovir molecules (Fig.2).

### Experimental

0.2 mmol ZnCl_2_ dissolved in 5 ml ethanol was added into 10 ml water containing 0.3 mmol pencicolvir. The mixture was stirred at room temperature for 5 h. The resulting solution was filtered. The filtrate was allowed to stay at ambient temperature for three weeks. Colourless block crystals were thus obtained. Yeild: 50%.

### Refinement

The water H and N(4) bound H were found from a difference Fourier map and refined freely. Other H atoms were treated as riding, with C—H distances of 0.97 and 0.98 Å,*N*—H distances of 0.86 Å, these hydroxyl O—H distances of 0.82Å and were refined as riding with *U*_iso_(H) = 1.2*U*_eq_ (C, N and O). Since the Flack value is 0(2) even after inverting the structure, the title compound is weak anomalous scatterer and therefore, Flack is meaningless.

### Crystal data

**Table:**

C_10_H_15_N_5_O_3_·H_2_O	*F*(000) = 576
*M**_r_* = 271.29	*D*_x_ = 1.438 Mg m^−^^3^
Orthorhombic, *P**n**a*2_1_	Mo *K*α radiation, λ = 0.71073 Å
Hall symbol: P 2c -2n	Cell parameters from 1193 reflections
*a* = 8.2020 (16) Å	θ = 3.4–25.2°
*b* = 13.889 (3) Å	µ = 0.11 mm^−^^1^
*c* = 11.001 (2) Å	*T* = 293 K
*V* = 1253.2 (4) Å^3^	Block, colorless
*Z* = 4	0.54 × 0.45 × 0.08 mm

### Data collection

**Table:**

Bruker SMART CCD area-detector diffractometer	1193 independent reflections
Radiation source: fine-focus sealed tube	1084 reflections with *I* > 2σ(*I*)
graphite	*R*_int_ = 0.075
ω scans	θ_max_ = 25.2°, θ_min_ = 3.4°
Absorption correction: multi-scan (*SADABS*; Sheldrick, 2001)	*h* = −8→9
*T*_min_ = 0.957, *T*_max_ = 0.994	*k* = −16→16
6830 measured reflections	*l* = −11→13

### Refinement

**Table:**

Refinement on *F*^2^	Primary atom site location: structure-invariant direct methods
Least-squares matrix: full	Secondary atom site location: difference Fourier map
*R*[*F*^2^ > 2σ(*F*^2^)] = 0.050	Hydrogen site location: inferred from neighbouring sites
*wR*(*F*^2^) = 0.118	H atoms treated by a mixture of independent and constrained refinement
*S* = 1.06	*w* = 1/[σ^2^(*F*_o_^2^) + (0.0604*P*)^2^ + 0.3*P*] where *P* = (*F*_o_^2^ + 2*F*_c_^2^)/3
1193 reflections	(Δ/σ)_max_ < 0.001
186 parameters	Δρ_max_ = 0.15 e Å^−^^3^
4 restraints	Δρ_min_ = −0.31 e Å^−^^3^

### Special details

**Table:**

Geometry. All e.s.d.'s (except the e.s.d. in the dihedral angle between two l.s. planes) are estimated using the full covariance matrix. The cell e.s.d.'s are taken into account individually in the estimation of e.s.d.'s in distances, angles and torsion angles; correlations between e.s.d.'s in cell parameters are only used when they are defined by crystal symmetry. An approximate (isotropic) treatment of cell e.s.d.'s is used for estimating e.s.d.'s involving l.s. planes.
Refinement. Refinement of *F*^2^ against ALL reflections. The weighted *R*-factor *wR* and goodness of fit *S* are based on *F*^2^, conventional *R*-factors *R* are based on *F*, with *F* set to zero for negative *F*^2^. The threshold expression of *F*^2^ > σ(*F*^2^) is used only for calculating *R*-factors(gt) *etc*. and is not relevant to the choice of reflections for refinement. *R*-factors based on *F*^2^ are statistically about twice as large as those based on *F*, and *R*- factors based on ALL data will be even larger.

### Fractional atomic coordinates and isotropic or equivalent isotropic displacement parameters (Å^2^)

**Table:**

	*x*	*y*	*z*	*U*_iso_*/*U*_eq_	
C1	0.0269 (5)	0.4750 (3)	0.0559 (4)	0.0321 (9)	
C2	−0.0398 (5)	0.3455 (3)	−0.0873 (4)	0.0354 (9)	
C3	−0.0027 (5)	0.3211 (3)	0.3070 (4)	0.0374 (10)	
H3	−0.0018	0.3037	0.3886	0.045*	
C4	−0.0242 (5)	0.3102 (2)	0.1084 (4)	0.0311 (9)	
C5	0.0189 (5)	−0.0152 (3)	0.2010 (4)	0.0375 (9)	
H5	−0.0741	−0.0176	0.1452	0.045*	
C6	0.0126 (5)	0.4027 (3)	0.1448 (4)	0.0323 (9)	
C7	−0.0735 (5)	0.1556 (3)	0.2279 (4)	0.0396 (10)	
H7A	−0.1567	0.1390	0.1689	0.048*	
H7B	−0.1185	0.1448	0.3083	0.048*	
C8	0.0713 (5)	0.0901 (3)	0.2102 (5)	0.0383 (9)	
H8A	0.1458	0.0977	0.2780	0.046*	
H8B	0.1286	0.1084	0.1366	0.046*	
C9	−0.0360 (6)	−0.0601 (3)	0.3195 (5)	0.0537 (13)	
H9A	−0.1190	−0.0197	0.3563	0.064*	
H9B	−0.0841	−0.1226	0.3032	0.064*	
C10	0.1551 (5)	−0.0729 (3)	0.1431 (4)	0.0424 (11)	
H10A	0.1837	−0.0435	0.0660	0.051*	
H10B	0.2504	−0.0699	0.1952	0.051*	
N1	−0.0334 (4)	0.2576 (2)	0.2150 (3)	0.0352 (8)	
N2	0.0253 (4)	0.4092 (2)	0.2691 (3)	0.0356 (8)	
N3	−0.0498 (4)	0.2759 (2)	−0.0038 (3)	0.0354 (8)	
N4	−0.0054 (4)	0.4399 (2)	−0.0607 (4)	0.0359 (8)	
N5	−0.0645 (5)	0.3259 (3)	−0.2040 (4)	0.0506 (10)	
H5A	−0.0871	0.2680	−0.2263	0.061*	
H5B	−0.0579	0.3710	−0.2573	0.061*	
O1	0.0604 (4)	0.56135 (18)	0.0709 (3)	0.0396 (7)	
O2	0.1144 (4)	−0.17160 (18)	0.1226 (3)	0.0485 (8)	
H2	0.0219	−0.1752	0.0948	0.073*	
O3	0.0950 (6)	−0.0711 (3)	0.4012 (4)	0.0760 (12)	
H3A	0.0878	−0.1234	0.4354	0.114*	
O4	0.1827 (4)	0.1915 (2)	0.5157 (4)	0.0518 (8)	
H4	−0.017 (7)	0.489 (4)	−0.119 (6)	0.068 (17)*	
H4A	0.243 (6)	0.234 (3)	0.549 (5)	0.069 (17)*	
H4B	0.222 (9)	0.1362 (19)	0.527 (8)	0.14 (3)*	

### Atomic displacement parameters (Å^2^)

**Table:**

	*U*^11^	*U*^22^	*U*^33^	*U*^12^	*U*^13^	*U*^23^
C1	0.036 (2)	0.029 (2)	0.031 (2)	−0.0005 (16)	−0.0003 (17)	−0.0003 (17)
C2	0.045 (2)	0.031 (2)	0.031 (2)	0.0021 (16)	0.0000 (18)	−0.0032 (19)
C3	0.050 (2)	0.032 (2)	0.030 (2)	0.0036 (17)	0.0007 (19)	0.0023 (18)
C4	0.040 (2)	0.0252 (18)	0.028 (2)	0.0044 (15)	−0.0008 (18)	−0.0019 (17)
C5	0.043 (2)	0.031 (2)	0.039 (3)	−0.0014 (16)	0.000 (2)	0.003 (2)
C6	0.041 (2)	0.0264 (18)	0.029 (2)	0.0027 (15)	−0.0015 (17)	−0.0031 (17)
C7	0.051 (2)	0.0270 (19)	0.041 (3)	−0.0016 (17)	0.0036 (19)	0.0063 (19)
C8	0.041 (2)	0.0288 (19)	0.045 (3)	−0.0016 (16)	−0.003 (2)	0.0041 (19)
C9	0.059 (3)	0.042 (3)	0.060 (4)	−0.004 (2)	0.014 (3)	0.006 (2)
C10	0.050 (2)	0.033 (2)	0.045 (3)	−0.0005 (17)	0.008 (2)	0.0043 (19)
N1	0.053 (2)	0.0217 (14)	0.031 (2)	0.0013 (13)	0.0017 (18)	0.0038 (15)
N2	0.0508 (19)	0.0308 (16)	0.025 (2)	0.0009 (15)	0.0000 (16)	−0.0018 (15)
N3	0.0492 (19)	0.0296 (17)	0.028 (2)	−0.0021 (15)	0.0012 (15)	−0.0013 (15)
N4	0.053 (2)	0.0274 (17)	0.028 (2)	−0.0031 (14)	0.0004 (16)	0.0017 (15)
N5	0.088 (3)	0.0347 (18)	0.029 (2)	−0.0071 (18)	−0.005 (2)	−0.0013 (16)
O1	0.0618 (18)	0.0260 (13)	0.0311 (18)	−0.0097 (13)	0.0054 (13)	−0.0011 (12)
O2	0.0553 (17)	0.0275 (13)	0.063 (2)	0.0027 (12)	0.0003 (17)	−0.0004 (15)
O3	0.109 (3)	0.066 (2)	0.053 (3)	−0.018 (2)	−0.018 (2)	0.0180 (19)
O4	0.0547 (18)	0.0418 (17)	0.059 (2)	0.0001 (16)	−0.0074 (17)	0.0026 (16)

### Geometric parameters (Å, °)

**Table:**

C1—O1	1.241 (5)	C7—C8	1.508 (6)
C1—N4	1.397 (6)	C7—H7A	0.9700
C1—C6	1.407 (6)	C7—H7B	0.9700
C2—N5	1.329 (6)	C8—H8A	0.9700
C2—N3	1.336 (5)	C8—H8B	0.9700
C2—N4	1.372 (5)	C9—O3	1.410 (6)
C3—N2	1.312 (5)	C9—H9A	0.9700
C3—N1	1.366 (6)	C9—H9B	0.9700
C3—H3	0.9300	C10—O2	1.429 (5)
C4—N3	1.340 (5)	C10—H10A	0.9700
C4—C6	1.379 (5)	C10—H10B	0.9700
C4—N1	1.383 (5)	N4—H4	0.94 (6)
C5—C9	1.514 (7)	N5—H5A	0.8600
C5—C10	1.515 (6)	N5—H5B	0.8600
C5—C8	1.528 (5)	O2—H2	0.8200
C5—H5	0.9800	O3—H3A	0.8200
C6—N2	1.375 (5)	O4—H4A	0.85 (5)
C7—N1	1.461 (5)	O4—H4B	0.84 (2)
			
O1—C1—N4	120.1 (4)	C7—C8—H8B	109.3
O1—C1—C6	128.0 (4)	C5—C8—H8B	109.3
N4—C1—C6	111.9 (3)	H8A—C8—H8B	108.0
N5—C2—N3	120.4 (4)	O3—C9—C5	111.5 (4)
N5—C2—N4	115.7 (4)	O3—C9—H9A	109.3
N3—C2—N4	123.9 (4)	C5—C9—H9A	109.3
N2—C3—N1	113.5 (4)	O3—C9—H9B	109.3
N2—C3—H3	123.2	C5—C9—H9B	109.3
N1—C3—H3	123.2	H9A—C9—H9B	108.0
N3—C4—C6	129.3 (4)	O2—C10—C5	113.7 (3)
N3—C4—N1	125.8 (3)	O2—C10—H10A	108.8
C6—C4—N1	104.9 (4)	C5—C10—H10A	108.8
C9—C5—C10	111.3 (3)	O2—C10—H10B	108.8
C9—C5—C8	114.9 (4)	C5—C10—H10B	108.8
C10—C5—C8	109.1 (3)	H10A—C10—H10B	107.7
C9—C5—H5	107.0	C3—N1—C4	106.1 (3)
C10—C5—H5	107.0	C3—N1—C7	126.6 (4)
C8—C5—H5	107.0	C4—N1—C7	127.3 (4)
N2—C6—C4	111.5 (4)	C3—N2—C6	104.0 (4)
N2—C6—C1	129.7 (4)	C2—N3—C4	111.5 (3)
C4—C6—C1	118.8 (4)	C2—N4—C1	124.6 (4)
N1—C7—C8	113.3 (3)	C2—N4—H4	122 (4)
N1—C7—H7A	108.9	C1—N4—H4	113 (4)
C8—C7—H7A	108.9	C2—N5—H5A	120.0
N1—C7—H7B	108.9	C2—N5—H5B	120.0
C8—C7—H7B	108.9	H5A—N5—H5B	120.0
H7A—C7—H7B	107.7	C10—O2—H2	109.5
C7—C8—C5	111.4 (3)	C9—O3—H3A	109.5
C7—C8—H8A	109.3	H4A—O4—H4B	110 (3)
C5—C8—H8A	109.3		
			
N3—C4—C6—N2	178.4 (4)	N3—C4—N1—C3	−178.5 (4)
N1—C4—C6—N2	−0.4 (4)	C6—C4—N1—C3	0.3 (4)
N3—C4—C6—C1	0.1 (6)	N3—C4—N1—C7	−0.5 (6)
N1—C4—C6—C1	−178.6 (3)	C6—C4—N1—C7	178.3 (3)
O1—C1—C6—N2	2.8 (7)	C8—C7—N1—C3	−98.2 (5)
N4—C1—C6—N2	−175.9 (4)	C8—C7—N1—C4	84.2 (5)
O1—C1—C6—C4	−179.3 (4)	N1—C3—N2—C6	−0.2 (5)
N4—C1—C6—C4	2.0 (5)	C4—C6—N2—C3	0.3 (5)
N1—C7—C8—C5	−169.4 (4)	C1—C6—N2—C3	178.4 (4)
C9—C5—C8—C7	−72.7 (5)	N5—C2—N3—C4	−178.8 (4)
C10—C5—C8—C7	161.5 (4)	N4—C2—N3—C4	0.8 (5)
C10—C5—C9—O3	56.0 (5)	C6—C4—N3—C2	−1.6 (6)
C8—C5—C9—O3	−68.6 (5)	N1—C4—N3—C2	176.9 (4)
C9—C5—C10—O2	56.1 (5)	N5—C2—N4—C1	−178.9 (4)
C8—C5—C10—O2	−176.0 (4)	N3—C2—N4—C1	1.4 (6)
N2—C3—N1—C4	−0.1 (4)	O1—C1—N4—C2	178.4 (4)
N2—C3—N1—C7	−178.1 (3)	C6—C1—N4—C2	−2.8 (5)

### Hydrogen-bond geometry (Å, °)

**Table:**

*D*—H···*A*	*D*—H	H···*A*	*D*···*A*	*D*—H···*A*
O2—H2···O4^i^	0.82	1.90	2.719 (3)	175
O3—H3A···N3^ii^	0.82	2.24	3.052 (3)	169
N4—H4···N2^iii^	0.96 (3)	1.86 (3)	2.816 (3)	176 (3)
O4—H4A···O2^iv^	0.86 (3)	1.93 (3)	2.787 (3)	178 (3)
O4—H4B···O1^v^	0.84 (3)	2.11 (5)	2.842 (3)	146 (3)
N5—H5A···O2^i^	0.86	2.15	2.898 (3)	146
N5—H5B···O1^iii^	0.86	2.11	2.931 (3)	159

Symmetry codes: (i) −*x*, −*y*, *z*−1/2; (ii) −*x*, −*y*, *z*+1/2; (iii) −*x*, −*y*+1, *z*−1/2; (iv) −*x*+1/2, *y*+1/2, *z*+1/2; (v) −*x*+1/2, *y*−1/2, *z*+1/2.
